# Endoscopic ultrasound‐guided choledochoduodenostomy versus hepaticogastrostomy combined with gastroenterostomy in malignant double obstruction (CABRIOLET_Pro): A prospective comparative study

**DOI:** 10.1002/deo2.70024

**Published:** 2024-10-06

**Authors:** Giuseppe Vanella, Roberto Leone, Francesco Frigo, Michiel Bronswijk, Roy L. J. van Wanrooij, Domenico Tamburrino, Giulia Orsi, Giulio Belfiori, Marina Macchini, Michele Reni, Luca Aldrighetti, Massimo Falconi, Gabriele Capurso, Schalk van der Merwe, Paolo Giorgio Arcidiacono

**Affiliations:** ^1^ Pancreatobiliary Endoscopy and Endosonography Division Pancreas Translational and Clinical Research Center IRCCS San Raffaele Scientific Institute Milan Italy; ^2^ Vita‐Salute San Raffaele University Milan Italy; ^3^ University of Turin Turin Italy; ^4^ Department of Gastroenterology and Hepatology University Hospitals Gasthuisberg University of Leuven Leuven Belgium; ^5^ Department of Gastroenterology and Hepatology Imelda General Hospital Bonheiden Belgium; ^6^ Department of Gastroenterology and Hepatology Amsterdam UMC University of Amsterdam Amsterdam Gastroenterology Endocrinology & Metabolism Amsterdam the Netherlands; ^7^ Pancreatic Surgery Unit Pancreas Translational and Clinical Research Center IRCCS San Raffaele Scientific Institute Milan Italy; ^8^ Medical Oncology Department Pancreas Translational and Clinical Research Center IRCCS San Raffaele Scientific Institute Milan Italy; ^9^ Hepatobiliary Surgery Unit IRCCS San Raffaele Scientific Institute Milan Italy

**Keywords:** cholangiopancreatography, cholangitis, endoscopic retrograde, gastric outlet obstruction, jaundice, stent

## Abstract

**Objectives:**

Malignant double obstruction, defined as the simultaneous presence of biliary and gastric outlet obstruction, represents a challenging clinical scenario. Previous retrospective experiences have demonstrated shorter dysfunction‐free survival (DyFS) of endoscopic ultrasound‐guided choledochoduodenostomy (EUS‐CDS) versus EUS‐hepaticogastrostomy (EUS‐HGS) in this setting, but no prospective evidence is available.

**Methods:**

Twenty consecutive patients with malignant double obstruction, treated with EUS‐gastroenterostomy (and EUS‐guided biliary drainage, following a previously failed ERCP, were enrolled in a prospective observational study (ClinicalTrials.gov NCT04813055) comparing EUS‐CDS versus EUS‐HGS. Efficacy and safety were evaluated, with Biliary Dysfunctions as the primary outcome and DyFS using Kaplan‐Meier estimates as a primary measure.

**Results:**

Twenty patients (75% with pancreatic cancer, 50% with metastatic disease) with EUS‐gastroenterostomy were included (seven EUS‐CDS and 13 EUS‐HGS). No significant difference was detected at baseline. Technical success was 100% in both groups. EUS‐CDS compared to EUS‐HGS showed similar clinical success (100% vs. 92.3%, *p* = 0.5), a higher rate of post‐procedural adverse events (42.9% vs. 7.7%, *p* = 0.067, mostly related to severe/fatal cholangitis in the EUS‐CDS group) and a higher rate of biliary dysfunctions during follow‐up (71.4% vs. 16.7%, *p* = 0.002).

DyFS was significantly shorter in the EUS‐CDS group (39 [15–62] vs. 268 [192–344] days, *p* = 0.0023), with a 30‐days DyFS probability of 57.1% vs. 100% (hazard ratio = 7.8 [1.4–44.2]).

**Conclusions:**

In this prospective comparison of patients with malignant double obstruction undergoing EUS‐gastroenterostomy, treating jaundice with EUS‐CDS versus EUS‐HGS resulted in a reduced probability of survival without biliary events and an increased risk of biliary dysfunctions (number needed to harm = 1.8), with detection of severe/fatal cholangitis.

## BACKGROUND

Jaundice and gastric outlet obstruction (GOO) are frequent manifestations of gastrointestinal malignancies which may negatively impact quality of life and delay oncological treatment. For GOO, growing evidence supports the use of endoscopic ultrasound‐guided gastroenterostomy (EUS‐GE) over duodenal self‐expandable metal stents (dSEMS).^1^, ^2^ Conversely, there is uncertainty regarding the optimal biliary drainage modality in this scenario, especially when endoscopic retrograde cholangiopancreatography (ERCP) fails. Whereas EUS‐guided biliary drainage (EUS‐BD) has proved superior over percutaneous drainage (PTBD),^3^ it is unclear whether EUS‐guided choledochoduodenostomy (EUS‐CDS) or EUS‐guided hepaticogastrostomy (EUS‐HGS) should be preferred.^4^

The combination of GOO and biliary obstruction (BO), or malignant double obstruction (mDO), represents a challenging clinical scenario since GOO might preclude access to the ampullary region and/or impair EUS‐BD, which relies on adequate gastroduodenal transit.^5^ Previous retrospective experiences suggested that EUS‐HGS results in less and later biliary dysfunction following either dSEMS^6^ or EUS‐GE^7^ but no prospective evidence is available. Furthermore, the risk of ascending cholangitis associated with EUS‐CDS in patients with duodenal obstruction has been reaffirmed by recent evidence from procedures involving lumen‐apposing metal stents (LAMS).^5^

To improve the reliability of these results, this study aimed to prospectively evaluate the long‐term outcomes of EUS‐CDS compared to EUS‐HGS in patients with mDO, where concomitant GOO was treated by EUS‐GE.

## METHODS

All consecutive patients treated for mDO in a single tertiary referral academic institution between July 2021 and October 2023 were enrolled in a Prospective Registry of Therapeutic EUS (PROTECT, ClinicalTrials.gov NCT04813055), with daily follow‐up during hospitalization and scheduled telephonic follow‐up every 30 days after discharge. Written informed consent was obtained for the procedures and, specifically, for the registry.

This prospective study was conducted in compliance with the Declaration of Helsinki and Good Clinical Practice and approved by the Ethics Committee (Id: 178/INT/2020).

This original cohort does not overlap with any previous publication, including the CABRIOLET retrospective study from the Leuven Amsterdam Milan Study Group.^7^

### Inclusion criteria

Patients were eligible upon receiving EUS‐GE for GOO and either EUS‐CDS or EUS‐HGS for biliary drainage after a failed or impossible ERCP.

Allocation to one of the two EUS‐BD modalities was not randomized but based on the performing endoscopist's judgment, taking into consideration the patient's clinical, morphological, and oncological characteristics.

Inclusion criteria were: (1) age >18 years; (2) ability to provide informed consent; (3) pathologically confirmed malignancy; (4) clinically and morphologically confirmed mDO; (5) having received endoscopic treatment with EUS‐GE plus either EUS‐CDS or EUS‐HGS; and (6) minimum post‐procedural follow‐up of 30 days, unless death occurred earlier.

mDO was defined as the simultaneous presence of malignant BO and GOO.

BO was defined as a radiologically and endoscopically confirmed biliary stenosis associated with jaundice (bilirubin ≥2 mg/dL).

GOO was defined as a radiologically and endoscopically confirmed gastroduodenal luminal stenosis associated with a GOO Scoring System (GOOSS^8^) score <2 (no food intake or ability to tolerate liquids only).

Exclusion criteria were: (1) benign BO or GOO; and (2) patients receiving biliary treatments other than those under evaluation, including EUS‐guided antegrade stenting.

### Procedures

**FIGURE 1:**
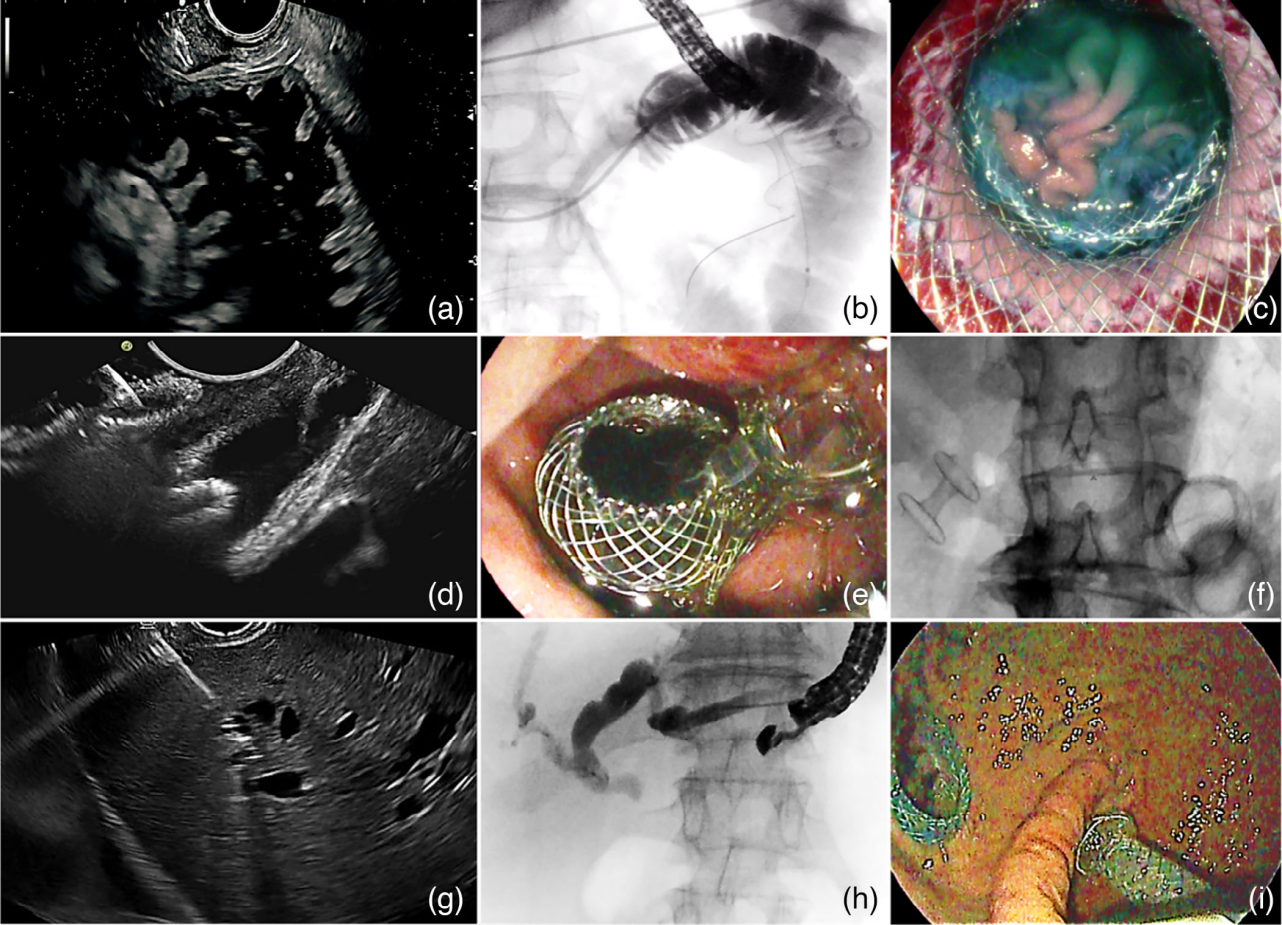
(a–c) Endoscopic ultrasound (EUS)‐guided gastroenterostomy (EUS‐GE). (a) Endosonographic appearance of the operative window for EUS‐GE before lumen‐apposing metal stents (LAMS) deployment (see the tip of the catheter in the left upper corner). The operative space includes an adequately distended jejunal loop, orientated in the direction of the operative channel of the scope, with the shortest distance possible between the gastric and jejunal lumen; (b) fluoroscopic image showing an oro‐jejunal tube looped in the first jejunal loop, through which the jejunum is distended (in this case with saline mixed with contrast) and a LAMS released between the stomach and the jejunal loop. (c) Visualization of the jejunal folds through the LAMS after stent release and dilation. (d–f) EUS‐guided choledochoduodenostomy (EUS‐CDS). (d) Endosonographic appearance of a LAMS placed between the choledochus and the duodenal bulb; (e) endoscopic image of a released proximal flange with bile flowing in the duodenum; (f) fluoroscopic image of a double obstruction managed by EUS‐GE + EUS‐CDS (with aerobilia). (g–i) EUS‐guided hepaticogastrostomy. (g) Endosonographic appearance of a 19G needle accessing a branch of a mildly dilated left biliary three through a cushion of hepatic parenchyma; (h) contrast injection confirming the puncture of a branch of the left biliary tree; (i) endoscopic appearance of a gastroenteric LAMS and of a hepatogastric SEMS.

EUS‐GE was performed (see Figure 1a–c) using the Wireless Simplified EUS‐GE Technique,^9^ involving an oro‐jejunal tube for jejunal distension and free‐hand placement of an electrocautery‐enhanced 20‐mm LAMS (Hot Axios; Boston Scientific).^1^

EUS‐CDS was performed (see Figure 1d–f) through the free‐hand placement of an 8 × 8 or a 6 × 8 mm LAMS (Hot Axios; Boston Scientific) between the common bile duct and the duodenum.^10^

EUS‐HGS was performed (see Figure 1g–i) by puncturing a left intrahepatic biliary branch through a 19G needle, followed by contrast injection, 0.035‐inch guidewire cannulation, tract creation using a 6Fr cystotome (Endoflex, GmbH), and placement of a 10 × 100 or 10 × 80 mm partially covered stent (Giobor; Taewoong).^10^

All procedures were performed by (or under the direct supervision of) two expert endoscopists (Paolo Giorgio Arcidiacono and Giuseppe Vanella) with extensive experience in therapeutic EUS (at least 50 procedures/year) and ERCP (at least 300 procedures/year) at the date of the first included patient.

### Outcome measures

The primary outcome of this study was biliary dysfunction, defined as the new onset of biochemical evidence of cholestasis or cholangitis accompanied by biliary dilation following initial clinical success, requiring hospitalization due to an escalation of medical care or biliary reintervention. The primary endpoints for this outcome were the rate (proportion) of biliary dysfunction^11^ and dysfunction‐free survival (DyFS), defined as the time from the procedure to the first occurrence of dysfunction, death, surgical resection, or the last clinical follow‐up, whichever occurred first. Prospective surveillance began on the day the two procedures (EUS‐BD plus EUS‐GE) coexisted, regardless of the order in which they were performed.

Technical success was defined as the successful completion of the planned procedures.

Among technically successful procedures, clinical success was defined as achieving a >50% reduction in bilirubin levels within 14 days after stent placement for BO,^11^ and a postprocedural GOOSS ≥2 (ability to eat at least soft solids) within 14 days after stent placement for GOO.^8^ Adverse events (AEs) were scored through the ASGE lexicon.^12^ Dysfunctions and reinterventions of EUS‐CDS were classified according to the Leuven Amsterdam Milan Study (L.A.M.S.) Classification.^5^

Procedural time (minutes) was limited to the interventional EUS‐BD fraction, ranging from the diagnostic EUS scan evaluating the target biliary structure to the completion of stent release.

### Statistics

Descriptive statistics are reported as frequencies (proportions) and medians (interquartile range) after the exclusion of normality. Comparisons between groups were performed by the Chi‐square or Fisher exact test for qualitative data and the Mann‐Whitney test for quantitative data, as appropriate.

Biliary dysfunction and DyFS were calculated using Kaplan‐Meier estimates, with censoring at the date of the last clinical follow‐up, death, or surgical resection (removing the biliary drainage), whichever came first. Log‐rank test was used to compare EUS‐CDS and EUS‐HGS. Due to the exploratory nature of this study, a formal sample size estimation was not conducted. A *p*‐value <0.05 was considered significant. All analyses were performed using Medcalc.

## RESULTS

During the study interval, 325 patients were included in the PROTECT Registry (see **METHODS**). Of these, 115 patients received EUS‐GE for GOO, 59 received EUS‐CDS, and 37 received EUS‐HGS.

For the scope of this study, 20 patients fulfilled the inclusion criteria, as they experienced mDO and underwent both EUS‐GE for GOO and EUS‐BD after failed/impossible ERCP: seven patients received EUS‐CDS and 13 EUS‐HGS.

### Baseline characteristics

Most patients (75%) had pancreatic cancer, and 50% had metastatic disease (see Table 1).

**TABLE 1:** Patient's characteristics of patients in the endoscopic ultrasound‐guided choledochoduodenostomy (EUS‐CDS) and hepaticogastrostomy (EUS‐HGS) groups.

Variable	EUS‐CDS, *n* = 7	EUS‐HGS, *n* = 13	*p*‐value
Age, years, median [IQR]	63 [61–76]	67 [60–74]	0.9
Male, *n* (%)	2 (28.6%)	9 (69.2%)	0.09
ASA score			0.7
2	5 (71.4%)	8 (61.5%)	
3	2 (28.6%)	5 (38.5%)	
Charlson Comorbidity Index, median [IQR]	4 [4–8]	8 [6–9]	0.08
Body mass index, kg/m^2^, median [IQR]	21.5 [18.4–24.4]	19.5 [18.6–23.4]	0.7
Primary disease			0.5
Pancreatic cancer	6 (85.7%)	9 (69.2%)	
Duodenal/ampullary cancer	1 (14.3%)	2 (15.4%)	
Others (gastric cancer/gallbladder cancer)	0	2 (15.4%)	
Oncological staging			0.3
Metastatic	3 (42.9%)	8 (61.5%)	
Localized	4 (57.1%)	5 (38.5%)	
Level of GI stenosis			0.3
Gastroenterostomy in post‐surgical anatomy	0 (0%)	3 (23.1%) *N* = 1 Roux‐en‐Y gastrectomy *N* = 2 Pancreatic surgeries	
Type 1 (above the ampulla)	3 (42.9%)	4 (30.8%)	
Type 2 (at the ampulla)	4 (57.1%)	6 (46.2%)	
Reason for EUS‐BD			0.4
Post‐surgical anatomy	0 (0%)	3 (23.1%)	
Papillary infiltration	5 (71.4%)	8 (61.5%)	
Failed ERCP	2 (28.6%)	2 (15.4%)	

Abbreviations: ASA, American Anesthesiological Association; ERCP, endoscopic retrograde cholangiopancreatography; EUS‐BD, EUS‐guided biliary drainage; GI, gastrointestinal; IQR, interquartile range.

No statistically significant difference between groups was detected at baseline. A non‐significant higher prevalence of post‐surgical anatomy was present in the EUS‐HGS group, together with a trend toward more advanced oncological staging (see Table 1).

EUS‐GE and EUS‐BD combinations were created with a median interval of 8 [0–38] days. Nine patients underwent same‐session double drainage (five EUS‐HGS; four EUS‐CDS). Six patients first received EUS‐GE, while five patients first received EUS‐BD, with a median latency of 36 [9–47] days. Of note, amongst patients with known GOO, the endoscopist tended to favor EUS‐HGS (83%), compared to the more balanced adoption of the two EUS‐BD modalities when BO was treated first (three EUS‐HGS in post‐surgical anatomy and two EUS‐CDS in normal anatomy).

### Outcomes

No patient was lost to follow‐up or had missing data for the outcomes of interest.

Technical and clinical success for EUS‐GE were 100% and 95% respectively, with a median pre‐ and post‐procedural GOOSS score of 0 [0–0] versus 2 [2–3], respectively.

Regarding EUS‐BD, technical success was 100% for both groups, with a significantly shorter procedural time for EUS‐CDS compared to EUS‐HGS (12.5 [4–15] min vs. 50 [34.5–58.5] min, respectively; see Table 2). The procedures showed similar clinical success (100% vs. 92.3%, *p* = 0.5).

**TABLE 2:** Outcomes of endoscopic ultrasound‐guided choledochoduodenostomy versus hepaticogastrostomy combined with endoscopic ultrasound‐guided gastroenterostomy.

Variable	EUS‐CDS, *n* = 7	EUS‐HGS, *n* = 13	*p*‐value
Technical success, *n* (%)	7 (100%)	13 (100%)	1
Median procedure time, minutes [IQR]	12.5 [4–15]	50 [34.5–58.5]	0.0006*
Clinical success, *n* (%)	7 (100%)	12 (92.3%)	0.5
Adverse events, *n* (%)	3 (42.9%)	1 (7.7%)	0.067
ASGE lexicon			0.5
Moderate	1 (14.3%) Cholangitis requiring reintervention	0	
Severe	1 (14.3%) Cholangitis requiring reintervention	1 (7.7%) Post‐ERCP pancreatitis (placement of retrograde SEMS in the right liver lobe)	
Fatal	1 (14.3%) Cholangitis complicated by septic shock and DIC	0	
Biliary dysfunctions^&^	5 (71.4%)	2 (16.7%)	0.02* RR = 4.3 [1.1–16.5] NNH = 1.8
Median hospital stay, days [IQR]	5.5 [5–19]	7 [5–11.8]	0.7
Median FU, days [IQR]	64 [27–102]	82 [22–217]	
Dysfunction‐free survival^$^			0.0023* HR = 7.8 [1.4–44.2]
Mean estimated DyFS, days (95% CI)	39 (15–62)	268 (192–344)	
DyFS probability			
30 days	57.1%	100%	
3 months	0%	87.5%	
Mean estimated OS^$^, days (95% CI)	105 (38–171)	133 (67–198)	0.9

Abbreviations: ASGE, American Society for Gastrointestinal Endoscopy; CI, confidence interval; DIC, disseminated intravascular coagulation; DyFS, dysfunction‐free survival; EUS‐CDS, endoscopic ultrasound‐guided choledochoduodenostomy; EUS‐HGS, endoscopic ultrasound‐guided hepaticogastrostomy; FU, follow‐up; NNH, number needed to harm; OS, overall survival; RR, relative risk; SEMS, self‐expandable metal stents

* Statistically significant.

^&^ Among patients with clinical success and ≥30 days of FU.

^$^ At Kaplan Meier estimator, with a log‐rank test.

However, a higher rate of post‐procedural AEs was noticed in the EUS‐CDS group (42.9% vs. 7.7%, p = 0.067). The three AEs in the EUS‐CDS group presented with septic shock due to cholangitis, occurring a median of 4 [2–7] days post‐procedure. One event was fatal, another required endoscopic reintervention with through‐the‐LAMS anterograde stenting due to stent obstruction, and the third was managed with antibiotic therapy. The only observed AE in the EUS‐HGS group was a moderate post‐ERCP pancreatitis in a patient receiving same‐session retrograde metal stenting of the right liver lobe, EUS‐HGS for the left liver lobe and EUS‐GE for duodenal invasion.

### Biliary dysfunctions

During follow‐up, the EUS‐CDS group experienced a significantly higher rate of biliary dysfunctions (71.4% vs. 16.7%, *p* = 0.02, relative risk = 4.3 [1.1–16.5]), resulting in a Number Needed to Harm of 1.8.

**FIGURE 2:**
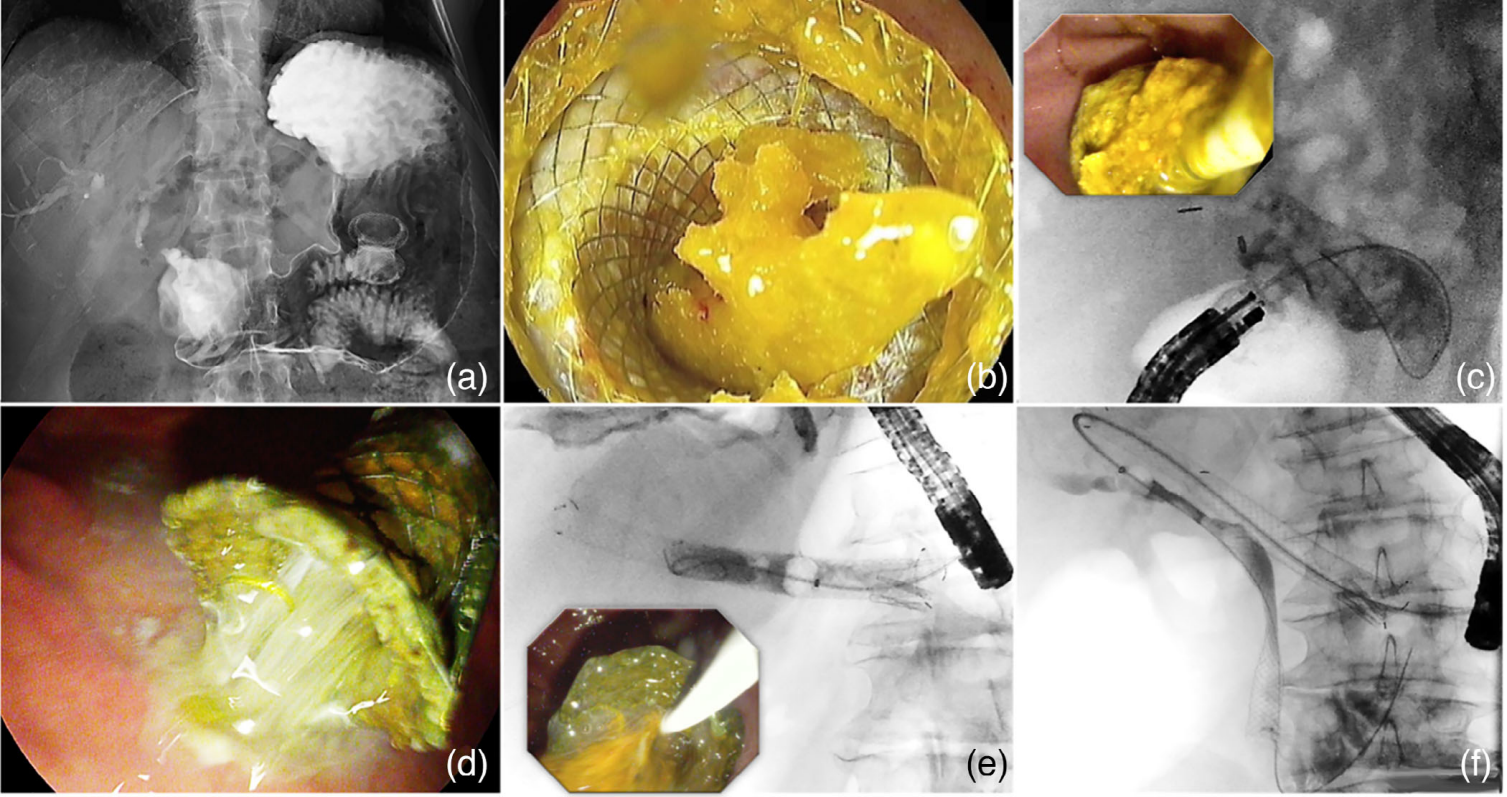
(a–c) Endoscopic ultrasound‐choledochoduodenostomy (EUS‐CDS) dysfunction. (a) During the follow‐up of a combination of EUS‐gastroenterostomy (EUS‐GE) + EUS‐CDS, the patient experienced cholangitis, and a gastrointestinal contrast series showed reflux of contrast medium inside the biliary tree through the lumen‐apposing metal stents (LAMS). (b) At endoscopic reintervention, food impaction was visible inside the choledocho‐bulbar LAMS; (c) balloon swipes were performed to extract food debris and sludge from the biliary duct. (d–f) EUS‐hepaticogastrostomy (EUS‐HGS) dysfunction. (d) During follow‐up of a combination of EUS‐GE + EUS‐HGS, the patient experienced recurrent jaundice and cholangitis. At endoscopic revision, the hepatogastric SEMS was visibly obstructed and showed an outflow of pus. (e) The SEMS was cannulated with a Fogarty balloon over the wire and balloon swipes were performed to clear accumulated debris; (f) at subsequent cholangiography, the end of the hepatogastric SEMS was angulated with respect to the direction of the left hepatic duct, and therefore biliary drainage was facilitated by anterograde placement of a transpapillary SEMS.

All dysfunctions in the EUS‐CDS group (*n* = 5) were related to ascending cholangitis from food impaction (type 5 according to the L.A.M.S. Classification,^5^ see Figure 2a–c). These were managed in one case conservatively with antibiotic therapy, in two cases with antegrade stenting through‐the‐LAMS (type D3 reintervention),^5^, ^13^ in one case with coaxial double‐pigtail stenting (type A reintervention),^5^, ^13^ and in another case with balloon swipes (type B reintervention).^5^, ^13^

The two dysfunctions in the EUS‐HGS group were managed in one case with balloon swipes and anterograde transpapillary stenting (see Figure 2d–f), while the other was managed conservatively with antibiotic therapy due to the patient's advanced stage of disease.

**FIGURE 3:**
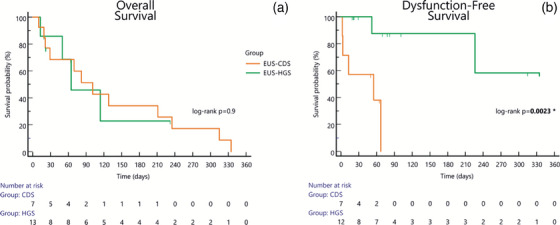
Kaplan‐Meier analysis of overall (a) and dysfunction‐free survival (b). (a) No difference in overall survival was noticed between the EUS‐choledochoduodenostomy (EUS‐CDS) and the EUS‐hepaticogastrostomy (EUS‐HGS) groups. (b) In the setting of combined biliary and alimentary obstruction, the latter treated by EUS‐gastroenterostomy, the EUS‐CDS group showed a significantly shorter survival without biliary events compared to the EUS‐HGS group (log‐rank test *p* = 0.0023).

At Kaplan‐Meier estimator (Figure 3b), EUS‐CDS in this setting showed a significantly shorter DyFS (39 [15–62] vs. 268 [192–344] days, log‐rank *p* = 0.0023, Hazard Ratio = 7.8 [1.4–44.2]), with a 30‐days DyFS probability of 53.6% vs. 100%. No major differences in median Overall Survival were detected (EUS‐CDS: 105 [38–171] days; EUS‐HGS: 133 [67–198] days, *p* = 0.9; Figure 3a).

## DISCUSSION

In this first prospective comparative study, EUS‐CDS exhibited a higher incidence of early cholangitis and a significantly shorter DyFS compared to EUS‐HGS, despite similar technical and clinical success rates. These data raise significant concerns about performing EUS‐CDS in case of double obstruction.

Managing mDO is complex. Duodenal obstruction can hinder ERCP or reduce the effectiveness of technically successful biliary drainage. To treat GOO, dSEMS have proved less effective, both in the short and long term, when compared to newer solutions such as EUS‐GE. In a recent meta‐analysis,^14^ EUS‐GE versus dSEMS demonstrated higher clinical success (94% versus 86%, pooled odds ratio (OR) = 2.72 [1.86–3.97, *I* ^2^ = 0]) and significantly lower need for reinterventions (4% versus 25%, OR = 0.13 [0.07–0.28], *I* ^2^ = 13]). These results are consistent even in prospective^1^ and randomized studies^2^ on this comparison. Notably, dSEMS had a higher AEs rate than EUS‐GE (20% versus 9%),^14^ despite EUS‐GE being perceived as a significantly more challenging procedure with a higher risk of misdeployment and perforation and a steeper learning curve.^15^ This might depend on the higher risk of bleeding of metal stents crossing a neoplastic infiltration, as well as on the increased risk of pancreatitis and cholangitis resulting when dSEMS are placed across the papilla,^16^, ^17^ either due to direct interference with biliary stents or increased duodenobiliary reflux. Finally, achieving biliary drainage through a dSEMS makes it more difficult to locate or access the papilla, or to place a biliary stent through the meshes; success rates for biliary drainage in retrospective studies vary between 34% and 93%, reflecting selection biases inherent in retrospective evaluations.^18^, ^19^, ^20^, ^21^

EUS‐BD is increasingly used in mDO. A recent study including 39 patients showed that EUS‐BD had higher technical (95% versus 56%; *p* < 0.01) and clinical success rates (91% versus 52%; *p* = 0.01) compared to ERCP, with similar invasiveness and long‐term patency.^22^ Additionally, comparing EUS‐HGS with ERCP or PTBD in the setting of indwelling DS, a trend towards lower complication rates and fewer reinterventions was observed for EUS‐HGS.^23^ Despite recent literature on mDO mostly focusing on patients already treated with DS, there is growing interest in procedural combinations involving EUS‐GE, due to the aforementioned advantages. The first study exploring this setting was an uncontrolled retrospective series (*n* = 23) describing same‐session EUS‐GE and EUS‐HGS, which showed high clinical success rates and a relatively low AEs rate.^24^

The L.A.M.S. Group published a multicenter retrospective study comparing different endoscopic combinations for treating mDO which suggested that combinations involving EUS‐GE achieve better long‐term outcomes compared to dSEMS and that either transpapillary stenting (via ERCP or antegrade stenting) or EUS‐HGS is associated with fewer symptom recurrences. Conversely, EUS‐CDS in this scenario showed lower primary success rates and frequent dysfunctions, regardless of whether GOO was managed by dSEMS or EUS‐GE.^7^ These data align with recent reports highlighting an increased risk of EUS‐CDS dysfunction in case of duodenal invasion or the presence of dSEMS.^5^, ^25^ Although EUS‐CDS might generally be preferred over EUS‐HGS for its simplicity and wider availability,^26^ our small prospective study confirms that EUS‐HGS significantly reduces the risk of short‐ and long‐term AEs in the setting of mDO, as it nullifies the risk of ascending cholangitis. All procedures were performed by endoscopists experienced in therapeutic EUS, and most AEs did not occur during the procedure but developed after a time interval, thus suggesting that they stem from EUS‐CDS's intrinsic limitations in this context rather than from operator inexperience or procedural errors.

Indeed, even after resolving GOO with EUS‐GE, the risk of food particles accumulating in the bulb where EUS‐CDS is positioned increases the likelihood of ascending cholangitis. This problem is potentially mitigated with EUS‐HGS, as the tubular stent design with long intragastric protrusion counteracts food influx; unlike the LAMS used in EUS‐CDS which reduces the interluminal distance between the duodenum and biliary tree.

This study has several limitations. First, despite the prospective inclusion, the allocation to EUS‐CDS versus EUS‐HGS was not randomized, and several factors might have influenced the endoscopist's decision. In some cases, EUS‐HGS was the only option in patients where the duodenum was not reachable (*n* = 4 non‐viable pyloric/bulbar stenosis; *n* = 3 post‐surgical anatomies); conversely EUS‐CDS might have been preferred in patients with more distal duodenal stenosis and viable duodenal bulb (*n* = 4) or in those where GOO was not yet clinically evident at the time of EUS‐BD (*n* = 3). Yet, there was a quota of patients in which the two procedures were both technically feasible, and one was chosen over the other, due to factors other than the anatomy, for example, potential future surgical candidacy, referring physician's preference, or simply lack of high‐quality evidence on the best EUS‐BD modality in this scenario. Moreover, there was a non‐significant temporal trend towards preferring EUS‐HGS in the latter part of the study period, due to accumulating evidence supporting this approach in the setting of concomitant GOO. Although all these factors might introduce a selection bias, baseline differences between the two groups, including the level of stenosis, were not statistically significant (see Table 1), but might become evident in a larger sample size. Nevertheless, regardless of the reasons that led to the preference for one technique over the other, we genuinely believe that this prospective series remains a unique opportunity to compare the patency of the two EUS‐BD modalities in this specific setting of double obstruction, where GOO was treated with EUS‐GE.

Secondly, in this series there was no routine adoption of prophylactic coaxial double‐pigtail plastic stents during EUS‐CDS: the impact of this maneuver on the risk of ascending cholangitis remains to be proven, and the results of a randomized controlled trial are eagerly awaited.^27^ Thirdly, these results were obtained from a tertiary referral center with significant expertise in both extrahepatic (EUS‐CDS) and intrahepatic (EUS‐HGS) biliary access, which may limit the generalizability of the findings. This last point prompts two distinct considerations. The first is that mDO represents a complex situation that might be better managed in tertiary referral centers, as standardized for complex surgery.^28^ The second is that whenever a center opts for EUS‐CDS and the problem of ascending cholangitis emerges, the choledochoduodenal LAMS can be exploited to perform rescue maneuvers, such as placement of coaxial double‐pigtail plastic stents or conversion to transpapillary stenting.^13^

Despite these limitations, this is the first prospective study comparing EUS‐HGS with EUS‐CDS in the setting of mDO treated exclusively by EUS‐GE. The prospective nature of post‐procedural follow‐up reduces the risk of underdetection of clinical events, even when managed in peripheral centers. In addition, no patient was lost to follow‐up, while the short follow‐up of this cohort simply reflects the reduced overall survival of patients with this condition, further highlighting the prognostic value of mDO.

To conclude, within the context of mDO, even when GOO is treated by EUS‐GE, EUS‐CDS reveals an unacceptably high risk of early and severe reflux cholangitis, a phenomenon seemingly less common with EUS‐HGS. To substantiate this proof‐of‐concept evidence, randomized controlled trials would be essential, though ethical considerations arise regarding the acceptability of exposing patients in the EUS‐CDS arm to such a heightened risk of biliary events. It remains to be seen whether universal prophylactic coaxial double‐pigtail plastic stents placement and further development of LAMS design will overcome the risk difference with EUS‐HGS, considering the easier execution and widespread availability of EUS‐CDS.

## CONFLICT OF INTEREST STATEMENT

Giuseppe Vanella reports lecture fees from Boston Scientific and travel grants from Euromedical. Michiel Bronswijk received grant support from Boston Scientific. Roy LJ van Wanrooij holds a consultancy agreement with Boston Scientific. Schalk van der Merwe has consultancy agreements with Cook Medical, Pentax, and Olympus and chairs the Boston Scientific board in Therapeutic Biliopancreatic Endoscopy and the Cook Medical board in Interventional endoscopy. All other authors declare no conflict of interest.

## ETHICS STATEMENT

Approval of the research protocol by an Institutional Reviewer Board: ID: **178/INT/2020**

Informed Consent: **Yes,** for the procedures, and for the observational prospective registry.

Registry and the registration no. of the study/trial: **ClinicalTrials.gov NCT04813055**

Animal Studies: **N/A**.
